# Improving recording of capacity to consent and explanation of side effects of medication in a large inner city service for adults – audit findings

**DOI:** 10.1186/1753-6561-6-S4-O6

**Published:** 2012-07-09

**Authors:** A Roy, S Jain, F Ward, C Richings, M Martin

**Affiliations:** 1 University of Manchester, UK

## Introduction

GMC Guidelines illustrate the importance of documenting key elements of a consultation such as side effects of medication and an assessment of patient's capacity. Not documenting this information makes a clinician liable if complications arise as there is no evidence of a discussion taking place. Do clinicians document this important information? Our aim was to look for evidence of:

1) An assessment of patients capacity to consent.

2) A discussion taking place regarding side effects.

This was from 6 Consultant teams. We also looked to evaluate the effect of measures introduced to improve practice.

## Methods

An audit was conducted in 2007, repeated in 2008 and 2009 using 156 case notes from each 6 Consultant team, looking for evidence of recording of the 2 sets of information. Results were given after each audit cycle to the Consultants, along with recommendations.

Following the re-audit in 2008 a rubber stamp was introduced prompting clinicians to record these discussions. The stamp was part of the service quality framework and a target of 90% adherence was set. Compliance with the stamp was recorded in the 2009.

## Results

Rates of recording of capacity rose from 30% in 2007 to 51% in 2009. (P=0.000006). Rate of recording of discussion about side effects was consistently higher than that of capacity showing little change between cycles, increasing from 71% in 2007 to 76% in 2009. The stamp was used in 60% of clinical encounters from 2008 - 2009. Capacity was more likely to have been recorded if the stamp was present (odds ratio 13.5 p<0.0001).

## Conclusions

The introduction of a stamp was associated with improvements in the recording of capacity to consent but had little effect on recording of discussions on side effects. This would suggest that the use of visual cues had improved compliance; clinicians became more efficient in documenting this important information. The audit was successful in improving practice within the 6 Consultant teams.

A possible limitation is that recommendations were given by medical students which may not have been taken as seriously as if given by a more senior body.

